# Full throttle: Demonstrating the speed, accuracy, and validity of a new method for continuous two-dimensional self-report and annotation

**DOI:** 10.3758/s13428-021-01616-3

**Published:** 2021-07-08

**Authors:** Kirill Fayn, Steven Willemsen, R. Muralikrishnan, Bilquis Castaño Manias, Winfried Menninghaus, Wolff Schlotz

**Affiliations:** 1 Max Planck Institute for Empirical Aesthetics, Frankfurt, Germany; 2 University of Groningen, Groningen, Netherlands

**Keywords:** Emotion dynamics, Affective dynamics, Momentary assessment, Two-dimensional continuous measurement, Continuous annotation, Continuous assessment

## Abstract

Research on fine-grained dynamic psychological processes has increasingly come to rely on continuous self-report measures. Recent studies have extended continuous self-report methods to simultaneously collecting ratings on two dimensions of an experience. For all the variety of approaches, several limitations are inherent to most of them. First, current methods are primarily suited for bipolar, as opposed to unipolar, constructs. Second, respondents report on two dimensions using one hand, which may produce method driven error, including spurious relationships between the two dimensions. Third, two-dimensional reports have primarily been validated for consistency between reporters, rather than the predictive validity of idiosyncratic responses. In a series of tasks, the study reported here addressed these limitations by comparing a previously used method to a newly developed two-handed method, and by explicitly testing the validity of continuous two-dimensional responses. Results show that our new method is easier to use, faster, more accurate, with reduced method-driven dependence between the two dimensions, and preferred by participants. The validity of two-dimensional responding was also demonstrated in comparison to one-dimensional reporting, and in relation to post hoc ratings. Together, these findings suggest that our two-handed method for two-dimensional continuous ratings is a powerful and reliable tool for future research.

**Supplementary Information:**

The online version contains supplementary material available at 10.3758/s13428-021-01616-3.

For research on people’s fine-grained and dynamic experiences of particular stimuli or situations, methods of *continuous self-report* are of crucial importance, as post-stimulus ratings cannot trace the dynamic trajectory of experiences over time. Accordingly, scholars have stressed the importance of continuous self-report measures in fields such as affective science (Ruef & Levenson, 2007), music studies (e.g., Geringer et al., 2004; Madsen, 1997; Schubert, 2010), affective computing (Cowie et al., 2000, 2012; Fuentes et al., 2017), communication studies (Biocca et al., 1994), organizational sciences (Gabriel et al., 2017), and interpersonal interactions (Lizdek et al., 2012; Sadler et al., 2009), as well as for annotation or observation purposes (Girard & Cohn, 2016).

Many research questions in psychology and other social sciences also involve relations between two or more experiential dimensions. Examples include studies on mixed emotions (e.g., Larsen et al., 2001; Larsen & McGraw, 2011, 2014), interpersonal dynamics (e.g., Hopwood et al., 2018; Ross et al., 2017), core affect (e.g., Madsen, 1996; Nagel et al., 2007), attitude ambivalence (e.g., Conner & Armitage, 2008), and emotion differentiation (e.g., Erbas et al., 2018). This makes accurate and valid methods for continuous self-report on two dimensions important for researchers across these fields.

Several methods have been developed to continuously track two dimensions of experience over time (e.g., Cowie et al., 2000; Girard & Wright, 2018; Larsen et al., 2009; Lizdek et al., 2012; Nagel et al., 2007). However, to our knowledge, no study has thoroughly investigated the validity of continuous ratings given for two dimensions at a time. Can we actually expect valid results if people are asked to attend to a stimulus, continuously assess two emotional states, and manually report on these at a time? Moreover, most available methods rely on reporting via a single joystick. This approach has the potential drawbacks of a) challenge of reporting on two states using one hand, and b) having the neutral state at the center of a two-dimensional space, which may be better suited for bipolar rather than unipolar constructs.

To address these possible limitations and provide a scientifically sound solution to open questions, the current study (a) proposes a method for continuous reporting on two dimensions of an ongoing experience that uses two hands, (b) compares the measurement reactivity, accuracy, speed and dimension independence of reporting to an existing method that tracks unipolar ratings (Larsen et al., 2009), and (c) examines the validity of two dimension continuous ratings by comparing rating profiles to single dimension ratings, as well as their relations to post-stimulus ratings. Methods are compared and validated using both visual (a short film and a feature film excerpt) and auditory (a poem) stimuli. Prior to presenting our new method, we briefly review in more detail the strengths and weaknesses of the methods of collecting continuous ratings as used to date, and describe our new method of dual-dimension continuous self-report.

## Current approaches to continuous measurement

Continuous self-report methods were pioneered in the 1930s (Peterman, 1940) and have come to be increasingly used from the mid-1980s onwards (see Biocca et al., 1994; Geringer et al., 2004; Gottman & Levenson, 1985; Gregory, 1989; Ruef & Levenson, 2007; Tan & van den Boom, 1992). Over the past two decades, multiple specialized tools and software packages have become available that support these methods (e.g., Cowie et al., 2000; Girard, 2014; Girard & Wright, 2018; Larsen et al., 2009; Nagel et al., 2007; Schubert, 1999; Sharma et al., 2017; Zhang et al., 2020). Moreover, statistical techniques have been developed that help to model the dynamics of experience based on continuous self-report data (e.g., Heylen, Van Mechelen, Fried, & Ceulemans, 2016a; Heylen, Van Mechelen, Verduyn, & Ceulemans, 2016b; McKeown & Sneddon, 2014; Suk et al., 2019).

Continuous ratings offer several advantages over *post-stimulus* ratings. The latter tend to capture either macro level (gist) data or micro-level (atomic) data on discreet segments (Girard & Wright, 2018) and are specifically useful for stimuli that do not by themselves unfold in time, such as standard picture libraries (e.g., Bradley & Lang, 2007), and typically only support short duration of exposure. At the same time, post-ratings offer no insights into important features of affective dynamics, such as the duration of an emotional episode, intensity profile shapes, emotional variability and instability, and emotional inertia (Kuppens & Verduyn, 2017). Importantly, continuous ratings do not appear to alter people’s subjective experiences as captured by retrospective ratings (Wagner et al., 2020).

Continuous ratings on two dimensions offer all of the advantages of single dimension continuous ratings, with the added advantage of being able to study and model the *dynamic interplay* of two states, without increasing the cognitive load compared to retrospective ratings (Zhang et al., 2020). Many fields are interested in such dynamics. For example, affective dynamics researchers investigate emotion covariation, emotional augmentation and blunting (Kuppens & Verduyn, 2017); music researchers are interested in the interplay of valence and arousal during music perception (Nagel et al., 2007; Schubert, 1999); and relationship researchers are interested in the varying balances of agency and warmth in couple interactions (Ross et al., 2017; Sadler et al., 2009).

## Limitations of current approaches

### Bipolar versus unipolar ratings scales

The majority of available tools for continuous reporting on two dimensions have been designed for studying *bipolar* constructs (e.g., Cowie et al., 2000; Girard & Wright, 2018; Lizdek et al., 2012; Nagel et al., 2007; Sharma et al., 2017; Zhang et al., 2020), *circumplex models* of affect (Russell, 1980) or the *interpersonal circumplex* (Horowitz et al., 2006; Lizdek et al., 2012; Sadler et al., 2009). These methods typically use joystick-based reporting, which place the neutral point in the center. However, in the field of emotion research, the assumption that valence is best represented as a bipolar construct has been topic of ongoing debate (cf. Dejonckheere et al., 2018; Larsen, 2017; Larsen & McGraw, 2011, 2014; Russell, 2017). For example, research on mixed emotions stipulates that some stimuli and situations evoke both positive and negative emotions (Larsen & Green, 2013; Larsen & McGraw, 2014). Similarly, research in aesthetics has shown that mixed appraisals of stimuli are quite common (Barford et al., 2018), and that emotional states such as ‘being moved’ (Menninghaus et al., 2015) or nostalgia (Barrett et al., 2010) involve coactivations of both positive and negative affect. Moreover, people's attitudes are at times ambivalent, i.e., involve both positive and negative evaluations of an object or an idea (Conner & Armitage, 2008). As shown by Larsen et al. (2009), such mixed states could be misleadingly reported as neutral when using bipolar ratings that do not allow for separate reporting on positive and negative dimensions of experience.

To address this issue, Larsen et al. (2009) developed the *evaluative space grid*, which allows to collect unipolar measures of positive and negative affect within one graphical grid and hence enables reporting and detection of mixed and ambivalent states. The neutral point is positioned in the bottom left corner (where both X and Y-values are at 0), and instead of a joystick—which has the neutral state in the middle of two dimensions—a mouse is used for reporting. Today, researchers across many fields are interested in the dynamic interplay of unipolar constructs, regardless of differences in their general stance towards the bipolar versus unipolar nature of affect. For all these researchers, the joystick method is problematic.

### Single-handed reporting

All previous methods for dual dimension reporting ask users to report the two evaluations with one hand. Typically, these methods require users to report the two dimensions by navigating a mouse or a joystick through a single coordinate on a grid. Such one-handed reporting may produce spurious relations between the two dimensions. For example, when a person wants to adjust their level of only one dimension—requiring moving a mouse or a joystick exactly vertically or horizontally—some movement on the other dimension is likely to also be recorded, thus introducing error variance on the second dimension. A possible solution, we propose, is the use of separate rating devices. Such devices may reduce spurious dependencies between the two reported variables.

### Graphical versus tangible user interfaces and attention

The evaluative space grid (Larsen et al., 2009) requires participants to report their evaluations by navigating a two-dimensional grid on a computer screen. For reporting on audio-visual stimuli, the screen has to be divided between the graphical grid and the media playback; this diminishes the size of the stimulus. Moreover, reporting by way of a graphical grid requires an at least partial visual focus on the grid and hence possibly involves saccadic eye movements between the grid and the media playback; this could detract from the absorbing or emotion-inducing qualities of the stimulus. Finally, using a mouse to report on experience has the disadvantage of providing limited tangible information to the participants because there are no physical limits to scrolling the mouse. As a result, participants have to visually keep track of how the movement of the mouse translates into an evaluation within the confines of the grid.

**Fig. 1:**
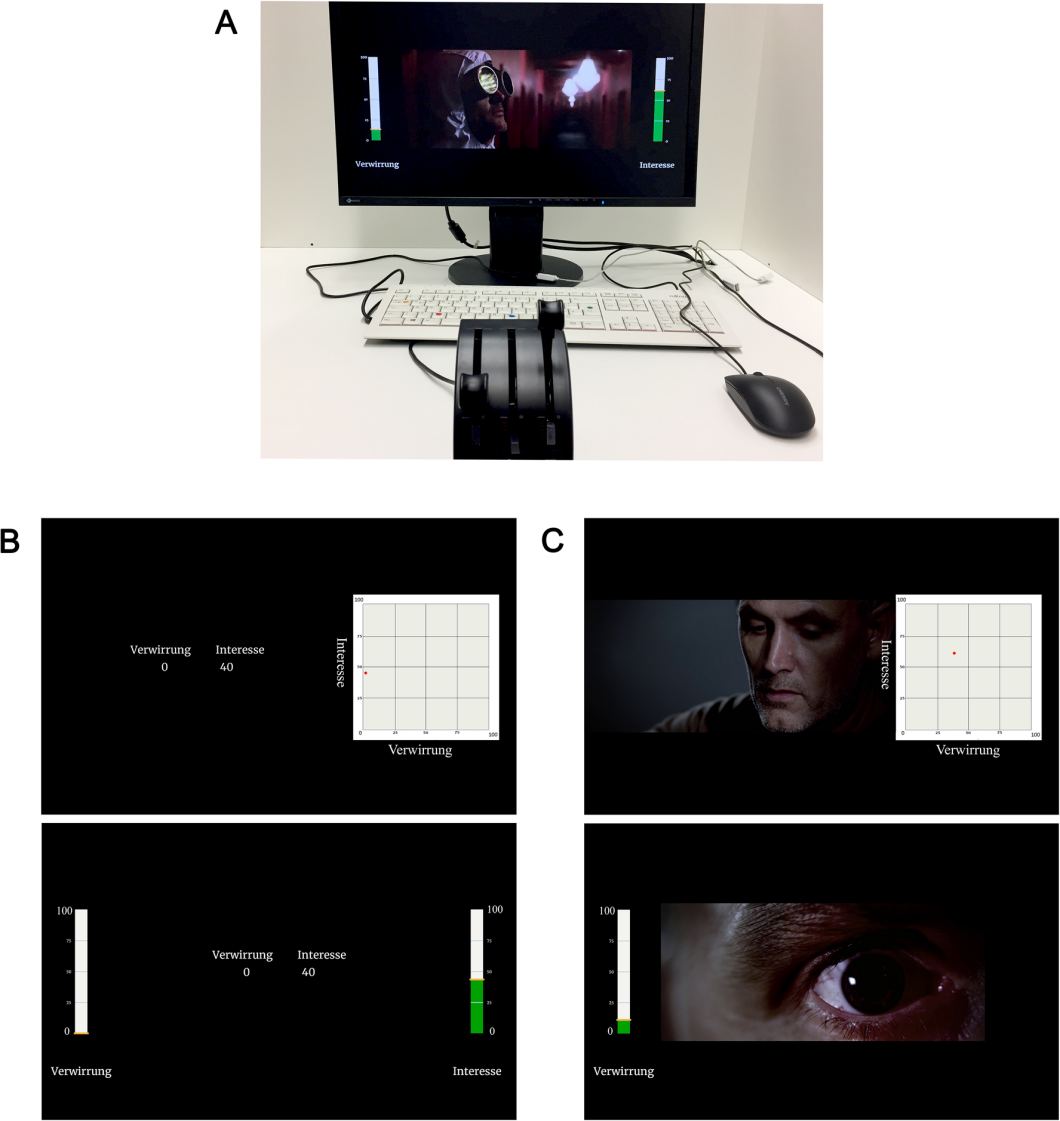
Experimental setup (**A**) and screenshots of the “follow the numbers” task (**B**), video responding with mouse and one throttle (**C**), and two throttles (**A**)

To address the above limitations, we propose an approach that separates the two unipolar dimensions both physically and visually. In line with a suggestion from Fuentes et al., (2017), we use a tangible user interface, specifically, physical sliders that provide more proprioceptive feedback, and thereby reduce reliance on visual confirmation. Visually, graphical representations of the two sliders were displayed on either side of a stimulus (see Figure 1 below). This approach has the advantage of being more salient—compared to a single dot on a two-dimensional grid—and requiring less saccadic movement between the stimulus and the response grid.

### Limited validation of two-dimensional measures

Previous validation of continuous two-dimensional measures has primarily focused on the methods’ utilities for annotation of couple behavior (Girard & Wright, 2018; Lizdek et al., 2012; Sadler et al., 2009), as well as annotation (Sharma et al., 2017; Zhang et al., 2020) and responses to affective stimuli (Larsen et al., 2009; Nagel et al., 2007). In annotation research, raters are trained for hours to detect and code behaviors (e.g., Sadler et al., 2009) in order to be consistent with other raters, resulting in impressive inter-rater reliability (Girard & Wright, 2018; Sadler et al., 2009). For responses to affective stimuli, images and videos are chosen that have previously been shown to reliably elicit a particular emotional state; the aggregated rater responses are then compared to previous post-stimulus ratings, or to theoretical expectations, to assess the validity of the continuous measures (Larsen et al., 2009; Nagel et al., 2007; Sharma et al., 2017; Zhang et al., 2020). Thus, in all of the examples described above, between-person consistency is the primary indication of validity, observed in experimental setups where consistency is influenced through either training or stimuli selected to target a particular state.

**Fig. 2:**
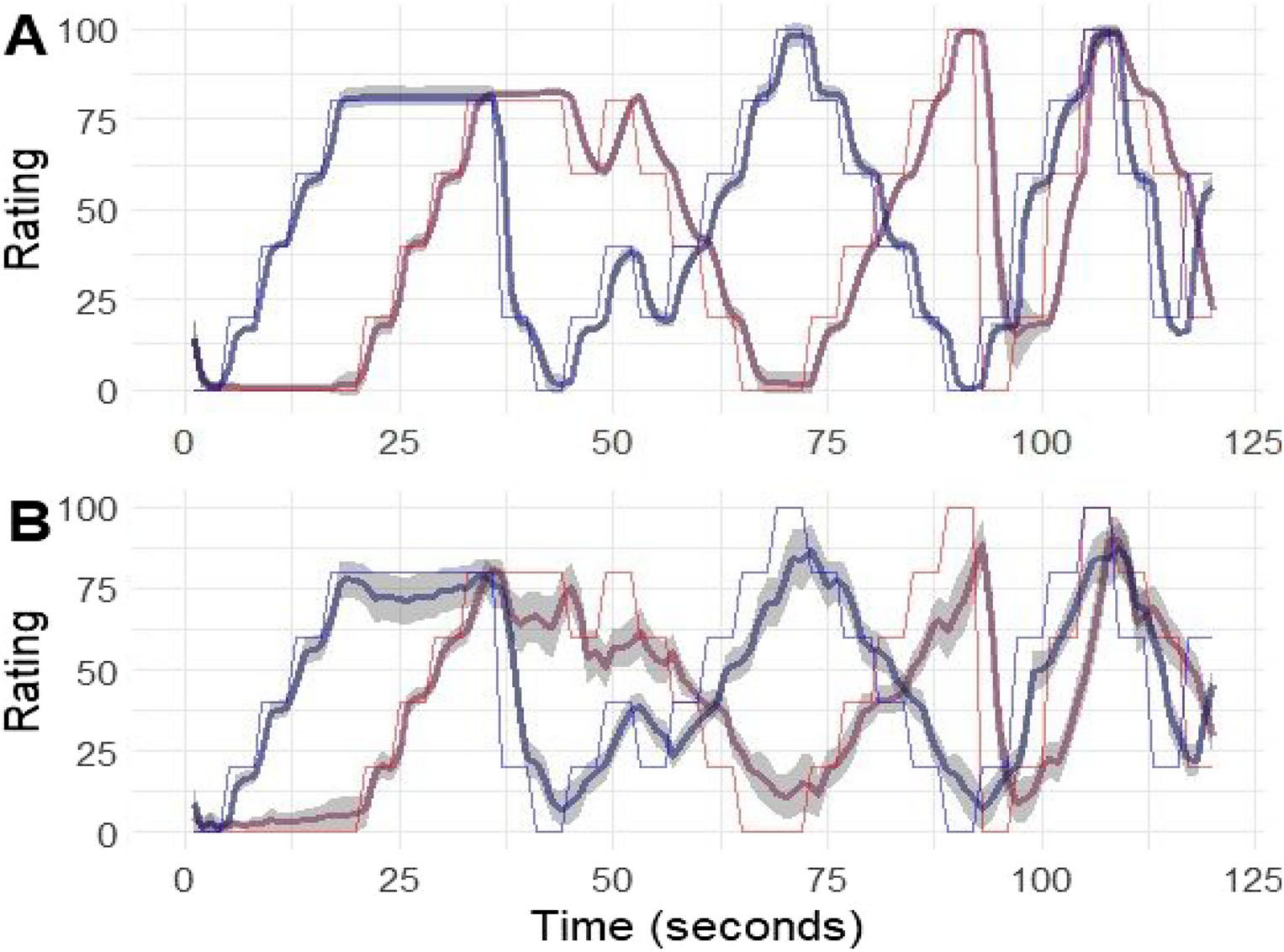
Target (*thin lines*) and observed data (*thick lines*) from the follow the numbers task for throttle (**A**) and mouse (**B**) conditions. The *grey-shaded regions* represent ± two standard errors around the mean

However, consistency is not the only goal of researchers interested in the dynamics of experience. In fact, many researchers are interested in experiences with stimuli that elicit idiosyncratic responses. For example, little consistency has been observed between participants that rated musical pieces (Nagel et al., 2007). However, Schubert (2012) reports strong within and between participant test-retest consistencies. Critically, consistency was observed in response to a relatively homogenous set of musical pieces (Romantic orchestral), with a sample that consisted primarily of participants with substantial music experience (Schubert, 2012)—which has been shown to lead to more consistent responding (Bigand et al., 2005)—while responses to pop and metal music are less consistent (Nagel et al., 2007). Additionally, substantial individual differences have been observed in response to movies chosen to elicit specific emotional experiences (Sharma et al., 2017; Figure 2). In sum, for experimental setups focusing on between-person consistency, there is sufficient evidence for the validity of continuous two-dimensional methods; for research focusing on idiosyncratic responses, such evidence is still lacking. One study provided such evidence where averaged continuous data was strongly related to post-stimulus ratings in response to musical pieces (Schubert, 1999). However, a purely auditory stimulus allows listeners to continuously focus on rating their experience, without the need to divert attention to a dynamic visual stimulus.

One further limitation of previous validation efforts is that none of the approaches described above have compared the validity of one-dimension continuous ratings to a two-dimensional approach. Such a comparison is important, as it tests the possibility that one-dimension ratings are more valid than the two-dimensional approach.

In summary, the current investigation aimed at extending the previously demonstrated validity of continuous two-dimensional ratings by a) comparing the validity of one versus two-dimensional responding, and b) by testing whether two-dimensional methods capture differences in experiences in response to stimuli that elicit more idiosyncratic responses, thereby validating such methods for the study of individual differences.

## The current study

The current study addressed the above concerns through two key research themes. First, by comparing our (two hand) method to the, to our knowledge, only other (single hand) method for continuous unipolar two-dimension reporting. Second, by testing the validity of *two-dimension self-report* data.

### Comparing single to two-hand reporting

We compared our approach to the *evaluative space grid* (Larsen et al., 2009) in terms of a) user preference, b) effects on mood, c) speed and accuracy of reporting, and e) independence of dimensions. We hypothesized that our method would address some of the limitations above by 1) allowing participants to report with two hands, 2) using two separate scales (rather than a single grid point) for rating indications, 3) placing visual representations of the current rating position (visual sliders) at the edges of the screen, thereby maximizing the visibility of the stimulus and limiting saccadic eye movements, and d) offering a more tangible rating device in the form of the physical sliders that have physical boundaries. Thus, we hypothesized that:

- H1: Participants prefer the throttle method to the mouse method in terms of ease-of-use ratings and head-to-head preference.
- H2: The effect of the throttle condition will not be associated with more negative mood, compared to the mouse condition.
- H3: The throttle method will be associated with more accurate and fast reporting compared to the mouse method.
- H4: The throttle method will have greater independence between the two dimensions compared to the mouse method.

### Usability and validity of dual versus single ratings

To determine the usability and validity of ratings on two dimensions, we compared them to single dimension ratings in terms of a) similarity of the profiles on the shared dimension, and b) participants’ self-reported ability to pay attention to a film stimulus during the rating task. Furthermore, we tested whether two-dimensional ratings are valid based on relations between profile features and post hoc ratings. Specifically, we tested whether dual dimension continuous ratings were valid in terms of adhering to the peak-end rule (Kahneman et al., 1993), which suggests that the best predictors of post experience ratings are the peak and end of the experience. Thus, our hypotheses were:

- H5: Participants will report similar ability to pay attention to stimuli between the one and two-throttles conditions.
- H6: Continuous profiles will be similar for the one- and two-throttles conditions.
- H7: The peak-end-rule will apply to continuous ratings on one and two dimensions.

## Method

### Participants

Power analyses were performed using G*Power. For repeated measures ANOVAs, a sample of 34 was required to detect a medium effect size (power = .8, α = .05). For correlation analyses, a sample of 29 was required (power = .8, α = .05), based on large effect sizes observed in the previous literature on the peak-end rule (e.g., Kahneman et al., 1993; Kemp et al., 2008). For between person ANOVAs, a sample of 52 was required to detect a large effect size (power = .8, α = .05). Therefore, sixty participants were recruited for the study. Due to an experimenter error, the age and gender information is not available for ten participants. For the 50 participants with available demographic data, the mean age was 37.24 (SD = 17.80), 22 were male, 27 female, and one participant indicated ‘other’ for gender. The study took about 45 min, and participants were compensated with 10 euros.

### Procedure

The full experiment consisted of four independent tasks aimed at testing the hypotheses and research questions outlined above. The study took part in a laboratory with a maximum of six participants per session. Before starting the experiment, all participants read an information statement and signed the consent form. Participants were then instructed, as a group, on the upcoming tasks. All tasks were preceded and followed by a mood questionnaire (Wilhelm & Schoebi, 2007), as well as some other questions regarding participant experiences (see full details in measures section). The whole experiment was programmed in Presentation software (Neurobehavioral Systems, Inc.).

### Experimental setup

We used the Logitech G Flight Simulator Throttle Quadrant to collect continuous ratings on two dimensions. The device is available from the Logitech website, as well as from various retailers across the world. The device connects to PCs via USB, and requires no adjustment for experimental purposes. For researchers using Presentation software, our experiment script is available from https://osf.io/t6uc4/, and the UI definition files are available from 10.5281/ZENODO.4032981. For researchers using other software, device integration should be straightforward for any software that recognizes USB plug-and-play response devices, such as Presentation, E-Prime, PsychToolBox, and PsychoPy.

Stimuli were presented on flat-screen displays with a resolution of 1920 x 1200 at 60 Hz refresh rate using the Presentation software (www.neurobs.com). A custom-made user interface (Muralikrishnan, 2019) was employed to program the experimental scenario. The physical coordinates of the mouse and throttle devices mapped on to appropriate UI elements on screen such that movements of the mouse changed the position of a moving circular pointer on a 2D grid canvas on screen, whereas throttle movements translated to virtual slider movements on screen. Although the devices were polled continually for their physical co-ordinates, a monitor refresh rate of 60 Hz meant that their position could be mapped on to changes on screen every 16.7 ms or higher. Device co-ordinates were thus recorded in intervals of 1–2 refresh rates (16.7–33.3 ms) with a timestamp resolution of 1/10th of a millisecond. Participants were seated approximately 40–50 cm in front of the monitor, and the mouse and throttle devices were ergonomically placed on the desk in front of them (Fig. 1A).

### Task 1: Follow the numbers - mouse versus throttle

The objective of this task was to compare the speed and accuracy of the mouse-based evaluative space grid method versus the two-hands throttle method (H3), the independence between the rating dimensions (H4), and to compare the methods in terms of measurement reactivity (H1, H2). The task involved using each input device to match two dynamically changing numbers on the screen. The numbers changed every 4 s and included blocks where change occurred a) on one dimension, b) on both dimensions in the same direction, c) on both dimensions in opposite directions, d) on both dimensions with the same magnitude of change, e) on both dimensions with different magnitudes of change (see the thin lines in Fig. 2 for these target profiles). Before starting the task, participants went through a 1-min training task—the same as the actual task, yet with a different target profile—to get used to using the input devices. The numbers and dimensions were (arbitrarily) labeled as ‘interest’ and ‘confusion’ to make sure the participants matched the numbers to the dimensions (see Fig. 1B). All participants completed both conditions—mouse and throttle—in a counterbalanced order.

### Task 2: Short film with state manipulation - one dimension versus mouse versus throttle

The objective of this task was to evaluate the sensitivity of the different methods in response to a strong signal (H5), and to compare the methods in terms of their effects on experiences with the stimulus (H6). A short movie scene (2:53 min) from *The Pursuit of Happyness* (2006) was manipulated to induce confusion^1^. This was achieved by flipping the film image upside down following an edit for 25 s during the scene. Participants were randomly assigned to three groups with 20 people per group. The first group rated their confusion using one throttle. The second group rated their interest and confusion using two throttles. The third group rated their interest and confusion using the mouse^2^. The comparison between the mouse and the two-throttles conditions allowed us to follow up on task 1 and test differences between two methods of giving two-dimensional ratings; the comparison between the single-throttle and the two-throttles conditions allowed us to test whether the addition of an extra rating dimension impacted reporting on the shared dimension (see Fig. 1A, C for screenshots of the three conditions).

### Task 3: One versus two dimensions in response to longer film

The objective of this task was to compare the validity of continuous ratings obtained by one versus two-dimensional continuous ratings for a longer stimulus^3^ (H6)—through investigating the respective relationships of these two continuous ratings with post hoc ratings (H6), and to compare the methods in terms of their effects on how the film was experienced (H6). Participants were randomly allocated to two groups of 30 people. The first group continuously reported on their confusion using one throttle, whereas the second group reported on their interest and confusion using two throttles.

### Task 4: Two-dimensional ratings in response to a poem

The objective of this task was to evaluate the validity of continuous ratings through the relationships with post hoc ratings. All participants listened to a professional recitation of the poem *Die Bürgschaft* by Friedrich Schiller (1798) and reported on their joy and sadness continuously using the two throttles. The poem has previously been used in research in our lab, and is known to elicit the state of being moved, which includes both joy and sadness components (Menninghaus et al., 2015; Wassiliwizky et al., 2017).

### Measures

#### Continuous measures

In the throttle conditions, participants continuously reported on their states on scales ranging from 0 to 100. Participants saw sliders on the screen, which had sub-divisions at 25, 50, and 75. In the mouse conditions, participants continuously reported on their states via a two-dimensional grid, again with in-between markers at 25, 50, and 75.

#### Mood scale

Between all tasks, participants reported on their mood on a validated six-item scale that assessed valence, calmness, and energetic arousal (Wilhelm & Schoebi, 2007). Participants reported on each scale via two bipolar items: valence was assessed using content-discontent and unwell-well items; calmness was assessed using agitated-calm and relaxed-tense items; energetic arousal was assessed using tired-awake and full of energy-without energy items. Across all tasks, limited differences between mood ratings across the conditions were observed (for details, see Supplementary Materials 1).

#### Post task measures

Following each mood scale, participants responded to several additional items after each task. Following both parts (mouse and throttle conditions) of task 1, participants responded to two bipolar items on a seven-point scale. The first item assessed the ease vs. difficulty of following the numbers on screen; the second item assessed the ease vs. difficulty of paying attention to the numbers during the task. These two items formed a scale with acceptable levels of reliability (ω_person_ = .71, ω_survey_ = .85). Following both conditions, participants were asked which of the two methods (mouse versus throttle) they found easier a) to follow the numbers and b) to pay attention to the numbers during the task.

Following tasks 2 and 3, participants responded to three items that asked to what extent the continuous rating task deflected their attention from actually experiencing the film. Specifically, participants were asked about the effect of the rating task on their a) distraction from, b) attention to, and c) experience of the film. The items were rated on a seven-point scale ranging from *not at all* to *very much*. The reliability of these three item scales was acceptable for both task 2 (ω = .73) and 3 (ω = .85). Due to the longer nature of the video in task 3, we further asked participants an extra item regarding the extent of tiredness that resulted from using the throttle(s). The item was rated on a seven-point scale ranging from *not at all* to *very much*.

#### Validation questions

Following tasks 3 and 4, participants rated the video (task 3) and poem (task 4) on aspects of experience relevant to the continuous ratings. All items were rated on a seven-point scale from *not at all* to *very much*. For task 3, participants reported on their overall interest and confusion in response to the film, and for task 4 participants reported on their overall joy, sadness, and being moved. Due to a computer error, interest, sadness, and joy post hoc ratings were only available for 39 participants.

### Statistical analyses

#### Task 1

Due to non-normality in the outcomes (see supplementary materials 2 for Shapiro–Wilk tests and normality plots for all conditions for tasks 1–3), differences in measurement reactivity between the two conditions were evaluated using a non-parametric analysis of repeated-measures via the f1.ld.f1 function from the nparLD package (Noguchi et al., 2012). Further, we tested the mixed effects model using robust statistical tests (Mair & Wilcox, 2019), specifically, via robust trimmed means mixed ANOVA (bwtrim function, trim = 0.2). For all robust group difference tests the Wilcox and Tian (2011) approach to effect size (ξ) was used.

The accuracy and speed differences between the two conditions were evaluated via a three-level growth model, in which observations are nested within conditions (mouse versus throttle), and conditions are nested within people. The dependent variable was the absolute deviation from target per person per second. At the within-person level, the absolute deviation was regressed on a time variable that coded for time since the last target change. Given that the targets were adjusted every 4 s, this variable varied from 1 (time of target) to 4 (four seconds since target change). Given that the first 4 s did not include any changes to the target, they were excluded from the analysis. This left 232 observations per person, divided into 29 four-second blocks per person per condition. At level two, the intercept and slope were regressed on the condition variable (with the throttle condition being coded as 0 and the mouse condition as 1).

To test whether the two methods differed in terms of amount of method-driven dependence, we selected parts of the task where changes occurred only on a single dimension, while numbers for the other dimension were static for that period. This was the case for the first 44 s of the task where only one of the dimensions changed (see thin lines in Fig. 1). Again, the first 4 s were excluded from the analysis since no target changes were present at the onset of these initial 4 s. Thus, per-person standard deviations were calculated for three time periods separately (4–20, 21–36, and 36–44 s). The standard deviations were then averaged per person across the three time periods to create an indicator of the standard deviations in responding where there should have been none. The variables of interest in both conditions were right skewed non-normal. We therefore tested the differences using robust statistical tests (Mair & Wilcox, 2019), specifically the robust test for two dependent groups (yuend function, trim = 0.2), and the non-parametric Sign test, since the distribution of differences were non-normal.

#### Tasks 2, 3, and 4

For task 2, the distribution of distractibility was right skewed non-normal for the one-throttle condition. For task 3, the distributions of distractibility and tiredness were right skewed non-normal for both conditions. For task 2, group differences were tested with the non-parametric Mood’s median test, since the distributions of the groups differed from each other. For task 3, differences were tested with the Mann–Whitney *U* test, since distributions were similar in shape. Group differences in both tasks were further tested with robust statistical tests (Mair & Wilcox, 2019), specifically the one-way trimmed means comparison (t1way function, trim = 0.2).

The validity of two-dimensional continuous ratings was determined by correlating the mean, peak, and end values from the continuous ratings with post hoc ratings for tasks 3 and 4. As some of the variables failed normality tests, we used Kendall’s Tau correlations as estimates of the relationships. To increase the robustness of these results, we also evaluated these relationships using robust percentage bend correlations (Mair & Wilcox, 2019), specifically the pbcor function.

## Results

The results are presented in the order of our two objectives. First, we present results regarding the utility, reliability, and measurement reactivity of the mouse versus throttle reporting methods. Second, results regarding the validity of single versus dual ratings are presented.

### Single versus dual hand rating methods

#### Task 1: Measurement reactivity—Ease of use and preference (H1)

Using the nonparametric analysis of repeated-measures, the difference in difficulty ratings between throttle (Mdn = 3.25, IQR = 2) and mouse (Mdn = 5, IQR = 1.5,) was significant, F(1, ∞) = 123.59, *p* < .0001, with the throttle condition being rated as less difficult. The influence on difficulty ratings was also different for the two orders of tasks, F(1, ∞) = 7.66, *p* = .006. Those that used the throttle first, reported greater difficulty overall. The interaction between method and order was not significant, F(1, ∞) = 0.64, *p* = .42).

The results using the trimmed mean tests were in line with the nonparametric tests, with significant differences for condition, F(1, 37.90) = 147.80, *p* < .0001, and order, F(1, 38.55) = 10.76, *p* = .002, but not the interaction, F (1, 33.32) < 0.0001, *p* > 0.999.

Finally, dichotomous ratings regarding preferences between the throttle and mouse conditions showed that 93.5% of participants preferred the throttle method. Together, these findings supported H1, suggesting that people strongly prefer the throttle sliders to the mouse, and found the throttle sliders easier to use.

#### Task 1: Accuracy and speed (H3)

The target profiles and observed data from the two conditions is presented in Fig. 2A is the throttle condition and Fig. 2B is the mouse condition. The faint lines represent the targets, and the thicker lines represent the aggregated observed data. The difference in accuracy between the two conditions is strikingly obvious from the figure, as is the difference in the variability between people—the error shades are almost unobservable in the throttle condition.

**Fig. 3:**
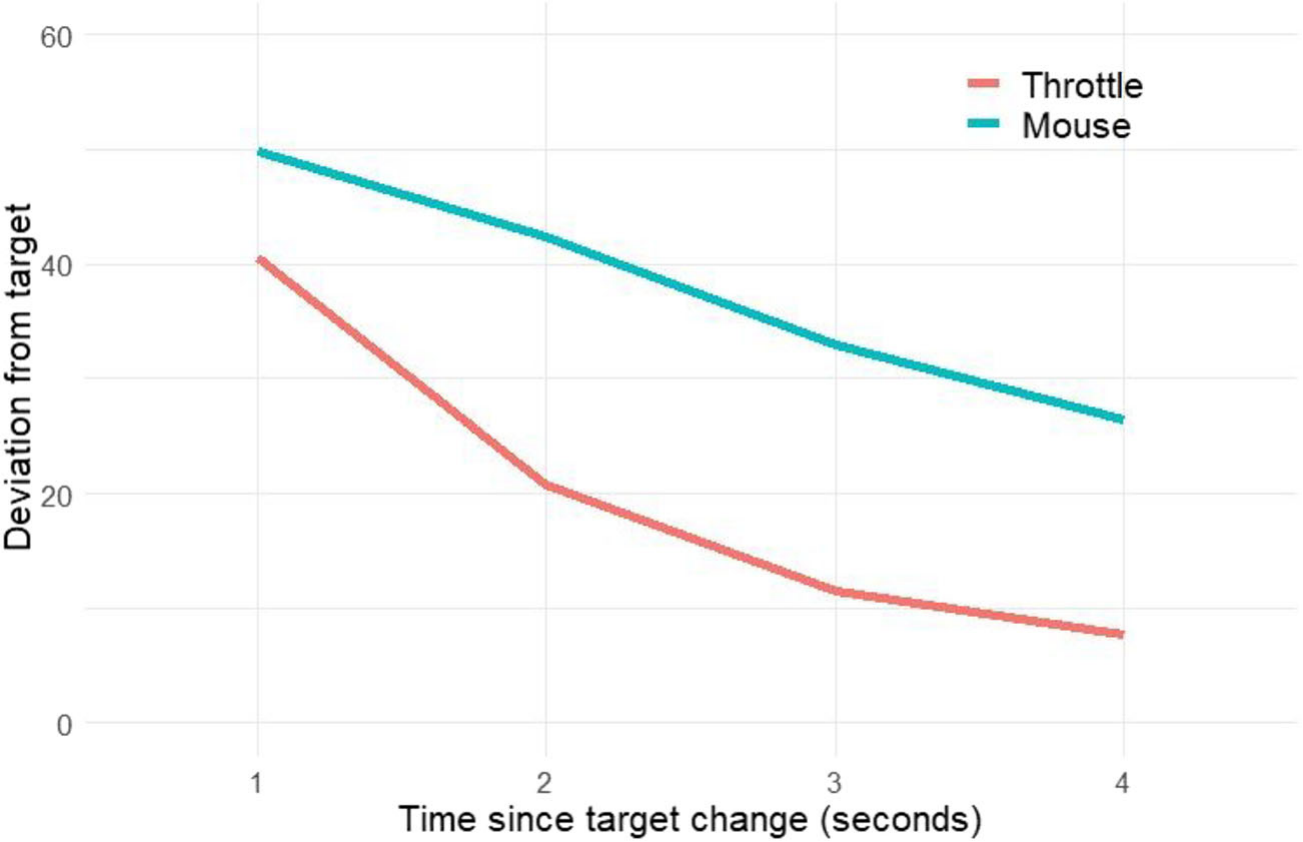
Average change in absolute deviation from target over time by condition

A three-level growth model was fit to test the difference between the two conditions. The level-one intercept was significantly higher for the mouse condition (b = 12.04, SE = 1.15, *p* < .001, 95% CI 9.79, 14.29]), indicating that at the time of the target change the throttle condition was closer to the target. The level-one slope was also predicted by the condition variable (b = 2.89, SE = 0.38, *p* < .001, 95% CI [2.14, 3.64]), suggesting that the throttle condition was associated with faster reduction of the deviation from the target. Together these findings suggest that the speed and accuracy of the throttle method was superior to that of the mouse condition. These findings can be seen graphically in Fig. 3, which plots the aggregated absolute deviation from the target in the 4 s from the change of the target. The deviation is the average of the absolute deviations from the targets on both dimensions.

#### Task 1: Independence between dimensions (H4)

Given that the primary goal of researchers collecting continuous ratings on two dimensions is to study the dynamics and relations between the two states, it is important that relations between the dimensions represent real relations rather than method-driven artefacts, which may result from using the same hand to respond on two dimensions.

Both the robust test, t(35) = 4.75, *p* = .00003, ξ = .77, and the Sign test, S(60) = 58, *p* < .001, suggested a significant difference in standard deviations for the mouse (Mdn = 4.68, IQR = 11.90) compared to the throttle (Mdn = 0, IQR = 0.43). According to the robust test, the explanatory measure of effect size was large. Notably, 81% of the sample had smaller SDs in the throttle condition, 52% of the participants had a standard deviation of zero in the throttle condition, and 88% of the participants had a standard deviation of less than 1. Conversely, only 18% of the participants in the mouse condition had a standard deviation of less than 1, and none had 0. This finding suggests substantial error variance in the mouse condition, whereas the error variance in the throttle condition was mostly negligible.

#### Task 2: Measurement reactivity differences between mouse and throttle conditions (H1)

The mouse condition (Mdn = 2.67, IQR = 1.83) was rated as the most distracting, followed by the two-throttles condition (Mdn = 3, IQR = 1.33) and the one-throttle condition (Mdn = 2, IQR = 1.75). However, these differences did not reach significance for the robust statistical test, F(2,21.4) = 2.89, *p* = .08, ξ = .38, nor for Mood’s median test, *p* = .15. Critically, this analysis was only sufficiently powered to detect large effect-size differences, thus suggesting the possibility of smaller, yet significant differences in future research, as suggested by the medium effect size observed.

#### Task 2: Profile differences between mouse and throttles (H5)

**Fig. 4:**
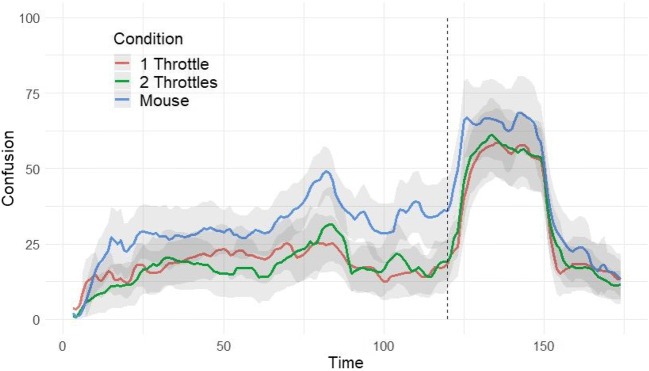
Confusion profiles by condition with manipulation marker. The *grey-shaded* regions represent ± two standard errors around the mean. The *dotted line* represents the confusion manipulation timing

The profiles for the shared dimension (confusion) of all three conditions in task 2 are presented in Fig. 4. The dotted line represents the point in time where the film image was flipped upside down (the confusion manipulation). As can be seen from the figure, the manipulation was effective in eliciting a response in all three conditions. The main difference that can be observed between the conditions is the level difference between the mouse and the two other conditions. This visual observation was tested by a multilevel random intercepts model. Confusion was regressed on two dummy coded variables that represented the mouse and two-throttles groups, therefore using the one-throttle group as a reference. Compared to the one-throttle condition, confusion was significantly higher in the mouse group, b = 10.85, SE = 5.10, *p* =.03, 95% CI [0.87, 20.84], but not in the two-throttles group, b = – 0.41, SE = 5.23, *p* = .94, 95% CI [– 10.67, 9.85].

### Validity of dual versus single dimension ratings

#### Task 3: Measurement reactivity of single versus dual dimension methods (H5)

Little distraction was reported in the one-throttle condition (Mdn = 2.17, IQR = 2.42,) and the two-throttles condition (Mdn = 2.17, IQR = 1.75,). The differences were not significant using the robust statistical test, F(1,31.38) = 0.008, *p* = .93, ξ = 0.02, nor with Mann–Whitney *U* test , *U* = 412.50, *p* = .58.

Ratings of tiredness were right skewed non-normal for both conditions. No significant differences were observed between the one-throttle (Mdn = 2, IQR = 2,) and two-throttles (Mdn = 3, IQR = 1,) conditions using the robust statistical test, F(1,33.59) = 0.08, *p* = .77, ξ = 0.06. Similarly, the Mann–Whitney *U* test revealed no significant differences, *U* = 466, *p* = .81. Together, these findings suggest that people do not report being adversely affected by the addition of a second dimension of rating.

#### Tasks 2 and 3: Similarity of profiles based on single versus dual dimensions (H6)

**Fig. 5:**
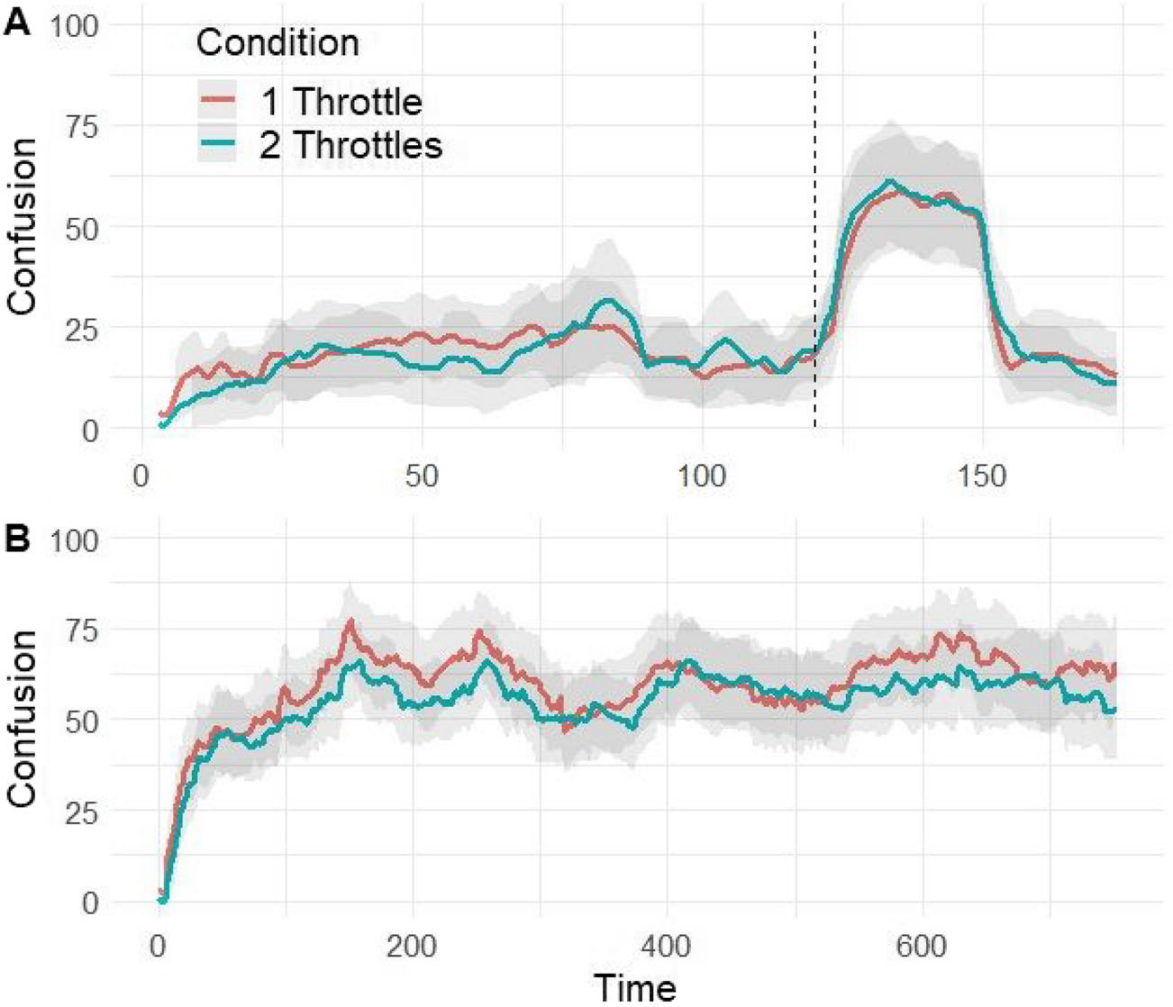
Confusion profiles for the one-throttle and two-throttles conditions. **A** The confusion profiles in responses to a short film with an experimental manipulation. **B** The confusion profiles in response to a longer film. The *grey-shaded regions* represent the 95% confidence interval around the mean. The *dotted line* in **A** represents the confusion manipulation timing

The profiles based on confusion ratings in a one-dimensional vs. a two-dimensional rating task are presented in Fig. 5. Visual inspection of the averaged profiles and their standard errors suggests that the profiles are very similar. The high overlap in the CIs suggests that the observable differences, if any, are negligible—not a single time point is significantly different between the two conditions—and that the response to the experimental manipulation is very similar for the two conditions.

#### Tasks 3 and 4: Predictive validity of dual dimension continuous ratings (H7)

As the results of the two types of analyses do not substantially differ, the robust correlations are reported in supplementary materials 3 (Tables S1, S2 and S3). The Kendall’s Tau correlations between post hoc confusion ratings and the mean, peak, and end confusion ratings are presented in Table 1. Correlations for the one-throttle group are above the diagonal, and below the diagonal for the two-throttles group. All correlations between post hoc confusion ratings and continuous profile features in the two-throttles conditions were significant and descriptively higher in the two-throttles condition. This suggests that the validity of the confusion ratings was not substantially affected by the inclusion of the second rating dimension.

**Table 1:** Kendall Tau correlations between post, mean, peak, and end confusion for the two conditions

	Post hoc confusion	Mean confusion	Peak confusion	End confusion
Post hoc confusion		.49**	.58**	.45*
Mean confusion	.51**		.52***	.52***
Peak confusion	.55**	.73***		.53***
End confusion	.68***	.59***	.55***	

Note: * *p* < .05; ** *p* < .01; *** *p* < .001; One-throttle condition correlations are displayed above the diagonal; Two-throttles condition correlations are displayed below the diagonal; Correlations between all variables and post hoc confusion ratings are based on 19 participants above the diagonal and 20 participants below the diagonal due to missing data. All other correlations are based on 30 participants.

The validity of the continuous ratings of interest—which were only available for the two-throttles condition—was similarly evaluated via correlations between the mean, peak, and end ratings and post hoc ratings (Table 2). Post hoc interest correlated positively with all three variables derived from the continuous ratings, but the relation with mean interest was not significant. Notably, this relationship, when converted to an estimate of effect size (Gilpin, 1993), represents a Cohen’s *d* of 1.23, suggesting a large effect size.

**Table 2:** Kendall Tau correlations between post hoc, mean, peak, and end interest

Variable	Mean interest	Peak interest	End interest
Post hoc interest	.35	.45*	.43*
Mean interest		.53***	.57***
Peak interest			.35*

Note: * *p* < .05; ** *p* < .01; *** *p* < .001; Correlations between post hoc interest ratings and all other variables are based on *n* = 20 due to missing data. All other correlations are based on *n* = 30.

##### Validity of poem post hoc ratings

Post hoc ratings of being moved correlated significantly with post hoc ratings of sadness (τ = .28, *p* = .03) and joy (τ = .53, *p* < .001). The correlations between continuous profile features and post hoc ratings are presented in Table 3. Post hoc sadness ratings correlated significantly with mean and peak sadness ratings, but not with the end ratings. The latter finding was to be expected, as the poem ends on high levels of joy and low levels of sadness. Thus, this lack of correlation actually supports the validity of the continuous sadness rating. Post hoc sadness also correlated significantly with the peak and end of the joy ratings. Post hoc joy ratings were strongly related to mean, peak, and end joy, but not with profile features for sadness. Post hoc ratings for being moved correlated with all profile features apart from end joy ratings. Together, these findings provide strong evidence for the validity of continuous joy and sadness ratings, indicating that two-dimension continuous self-reports provide highly valid data.

**Table 3:** Kendall’s Tau correlations between post hoc joy, sadness and being moved, and sadness and joy profile features

Variables	Sadness			Joy		
	Mean	Peak	End	Mean	Peak	End
Post hoc sadness	.40***	.36**	.08	.12	.29*	.29*
Post hoc joy	.23	.16	.11	.36**	.41***	.47***
Post hoc moved	.26**	.25*	.08	.19*	.32***	.28**

Note: * *p* < .05; ** *p* < .01; *** *p* < .001; Correlations between post hoc sadness and joy are based on 39 people. All other correlations are based on 60 people.

## Discussion

The study reported here developed a method for two-dimensional continuous self-report that addressed several potential limitations inherent to currently used methods. We compared the new method to a previously developed method by comparing rating profiles for one vs. two dimensions, and we evaluated the validity of two-dimensional ratings by testing whether or not both dimensions adhere to the peak-end rule (Kahneman et al., 1993).

In comparison to the mouse method, the throttle method was rated as easier to use and was strongly preferred by the participants. The throttle method was also associated with faster and more accurate responding. Moreover, we demonstrated that the mouse method might produce spurious relationships between the two dimensions, whereas we obtained no such indications for the throttle method. In our view, methods that rely on responding on two dimensions with one hand are more likely to be subject to a method-driven confounding of the two dimensions under scrutiny than our two-handed method. This assumption could be further tested by comparing the method presented here with single-handed joystick-based approaches to collecting two continuous ratings at a time (e.g., DARMA; Girard & Wright, 2018).

Regarding the validity of two-dimensional responding, profiles derived from single-dimension ratings were very similar to those obtained through continuous ratings on two dimensions—suggesting that two-dimensional ratings have similar validity of experience on average. Moreover, continuous ratings on two dimensions were valid in terms of their relations with post hoc ratings of auditory and visual stimuli—going beyond previous validation efforts that have primarily sought consistency between people’s responding.

### Mouse versus throttle versus joystick

In this study, we compared our full-throttle method to the mouse method. In previous research, joysticks have also been used for collecting continuous two-dimensional self-reports. While we are not able to directly compare either method used in this study to the joystick, we believe that methods using different kinds of input devices all have their strengths and weaknesses depending on the particular aim of an investigation. Thus, several factors should be considered before choosing a device.

The type of constructs of interest may influence the choice of device. For unipolar constructs, the neutral point is the zero point on both dimensions. Since the joystick has its neutral point in the center, the mouse or throttle may be more appropriate. However, given other potential advantages of the joystick over the mouse—such as clear end points and a more ergonomic grip—a possibility for future research lies in exploring the use of only one quarter of the joystick’s possible field of motion (upper right) for reporting on unipolar constructs. Such an approach could also have limitations: the range of movement would be restricted, and the scale would not have clear zero points on the *x*- and *y*-axes. Another alternative is the possibility to turn off the centering function on certain models of joystick. However, this approach would arguably take away some of the intuitiveness of the device. Critically, both of these adjustments would, arguably, not address the possibility of a lack of independence between the two dimensions that we observed in the mouse method. This possibility warrants further exploration.

For bipolar constructs, the joystick provides several advantages over the mouse and throttle methods. First, the joystick has its natural resting point in the center of a two-dimensional space—the neutral state or a value of zero on both dimensions—making the setup very intuitive for bipolar constructs. Second, the joystick provides ongoing tactile feedback regarding its position through a force that pushes it back towards the neutral point. While this is frequently mentioned as an advantage of the method (e.g., Nagel et al., 2007; Sadler et al., 2009; Sharma et al., 2017), it is not clear how consciously aware participants are of differences in the magnitude of the force, particularly when the force is felt in two dimensions. Further, the force may also bias measurements towards greater reporting of neutral states. For example, Sharma et al. (2017) found that reporting was particularly concentrated around the center of the valence-arousal space. This could be indicative of the prevalence of neutral states, but it could also result from the bias that pushes the joystick towards this state. Future research might aim at disentangling these two potential explanations.

Finally, when comparing the speed and accuracy of input devices in pointing to a specific point in a two-dimensional space, the mouse has consistently outperformed the joystick (Epps, 1986; MacKenzie et al., 2001; Pedersen et al., 2020; Ramcharitar & Teather, 2017). Thus, researchers that wish to utilize the combination of continuous dimensions along with several discrete states within the two-dimensional space, may still wish to use the mouse as an input device due to its greater speed and accuracy.

## Limitations and future directions

While our findings are overall very clear, several limitations of our study should be noted. We used unvalidated subjective ratings to gauge differences in attention and tiredness, whereas more objective methods such as eye tracking (Korbach et al., 2018) or validated cognitive load measures such the NASA-TLX (Hart & Staveland, 1988) are available. Zhang et al. (2020) used these measures to show that continuous two-dimensional self-reports did not lead to greater cognitive load compared to retrospective reporting. Since our study showed an almost exclusive preference for the throttle, with a large effect size, in the only within-person part of our study—arguably the most valid comparison—we feel confident in concluding that our method is strongly preferred to the mouse method. However, future studies seeking to illuminate the physical and psychological processes underlying this preference should ideally proceed using validated cognitive load measures.

Second, no direct comparison was possible between our method and the joystick method within this study. Future research may well show that the limitations we demonstrate for the mouse do not apply to the joystick. As we have proposed above, the spring action that returns the joystick to the neutral position may have costs and benefits, and the possibility of dimension independence—that was almost absent with our method—could be a limitation of the joystick. Thus, a direct comparison is called for. One possibility for such a comparison is to adjust the throttle method to measure bipolar constructs. This is readily doable, and differences between the two methods with regard to bipolar constructs could thus be directly evaluated.

Third, another interesting development in continuous two-dimensional measurement is its application in measuring experiences in virtual reality (cf. Xue et al., 2021). Within this context, too, the joystick method may be of limited value for measuring unipolar constructs, and because fine-grained movement along a single dimension may be more difficult with a small joystick such as the one integrated into an Oculus controller. Nonetheless, unipolar constructs could be measured by following a method comparable to our throttle, using two controllers and limiting the reporting to movement in one direction (e.g., moving each joystick up to indicate experiences of the unipolar constructs). Additionally, it may be worth exploring our method in the context of mobile rating devices too. Mobile phone applications could replicate our interface through touchscreen sliders on both ends of the screen, thus allowing for a continuous measurement of two unipolar constructs while attending to audiovisual material.

## Conclusions

In sum, our findings suggest that two-handed reporting that uses tangible as well as visual user interfaces greatly improves the usability, speed, and accuracy of two-dimensional self-reports, compared to the mouse method. This new approach therefore has the potential to minimize the method-driven user errors that we observed with a previously developed measure. Further, the current study suggests that profiles derived from two-dimensional ratings are equally valid indicators of individual differences in experiences compared to one-dimensional measures.

These results have potential for improving the measurement of dynamic psychological processes in future studies. Given the slider and throttle devices’ relatively low cost and compatibility with various experimental software, the two-handed method should be relatively easy to implement. The method presented here should be useful across several fields such as affective dynamics (e.g., Kuppens & Verduyn, 2017), mixed emotions (Larsen & McGraw, 2014), relationship and interpersonal interaction research (Hopwood et al., 2018; Ross et al., 2017), attitude ambivalence (Conner & Armitage, 2008), music studies (Schubert, 2010), and affective computing (Fuentes et al., 2017), as well as for purposes of observation and annotation (Girard & Cohn, 2017).

## Supplementary Information

ESM 1 (DOCX 316 kb)

## Data Availability

The data and experimental script are available from https://osf.io/t6uc4/.

## Footnotes

1 Confusion was chosen because a) several of the authors have experience with this construct, b) the project served as a validation of the method for a future experiment on interest-confusion dynamics, and c) it is a unipolar construct that is relatively easy to manipulate by flipping the screen.

2 Interest and confusion are examples of unipolar constructs that are related to each other to a degree, but not enough to be considered as a bipolar construct (Fayn et al., 2019).

3 The short film *Mouse-X* (2014) by Justin Tagg (12:53 min).

## Declarations

### Code availability

The code is available from https://osf.io/t6uc4/.
