# Supplementary material for: Full throttle: Demonstrating the speed, accuracy, and validity of a new method for continuous two-dimensional self-report and annotation
Source: Behav Res Methods. 2021 Jul 8;54(1):350–64. doi: 10.3758/s13428-021-01616-3 (PMC8863702; doi:10.3758/s13428-021-01616-3)
Supplement: Supplementary file 1 — (DOCX 316 kb) [file 13428_2021_1616_MOESM1_ESM.docx]

**Supplementary materials 1 - Mood differences between conditions**

**Mood scale reliability**

McDonald’s Omega (ω) was calculated as a metric of reliability using multilevel confirmatory factor analysis (Geldhof, Preacher, & Zyphur, 2014). The scales all had acceptable levels of reliability - calmness (ω_within_ = .85, ω_between_ = .77), valence (ω_within_ = .88, ω_between_ = .78), and energetic arousal (ω_within_ = .91, ω_between_ = .67).

**Mood effects Task 1**

Differences in calmness, energetic arousal and valence were evaluated using repeated measures ANCOVAs. For all mood scales, the mouse versus throttle condition variable was the within-person factor, the condition (order) variable was used as a between person variable, and the baseline mood ratings were used as a covariate.

For calmness, participants in the throttle condition (M = 4.24, SD = 1.10) reported greater calmness compared to the mouse condition (M = 3.78, SD = 1.20), F(1,57) = 5.34, *p* = .02. There was no significant effect of order of task, F(1,57) = 0.24, *p* = 0.63, nor was there a significant interaction between condition and order, F(1,57) = 0.02, *p* = .89. Participants that reported lower calmness before the task also reported lower calmness after the tasks, F(1,57) = 19.91, *p* < .001.

For energetic arousal, participants in the throttle condition (M = 4.62, SD = 1.13) reported similar arousal compared to the mouse condition (M = 3.88, SD = 1.28), F(1,57) = 0.005, *p* = .94. There was no significant effect of order of task, F(1,57) = 0.14, *p* = 0.71, nor was there a significant interaction between condition and order, F(1,57) = 0.97, *p* = .33. Participants that reported lower arousal before the task also reported lower arousal after the tasks, F(1,57) = 80.01, *p* <.001.

For valence, participants in the throttle condition (M = 4.28, SD = 1.13) reported similar valence compared to the mouse condition (M = 4.07, SD = 1.27), F(1,57) = 0.62, *p* = .43. There was no significant effect of order of task, F(1,57) = 2.11, *p* = 0.15, nor was there a significant interaction between condition and order, F(1,57) = 1.71, *p* = .20. Participants that reported more positive valence before the task also reported more positive valence after the tasks, F(1,57) = 19.33, *p* <.001.

**Mood effects Task 2**

Differences in mood (calmness, energetic arousal and valence) were evaluated using between-participant ANOVAs. For all mood scales, the condition variable was the fixed factor, and the baseline mood ratings (mood following previous task) were used as a covariates.

For calmness, participants in the one throttle condition (M = 5.03, SD = 0.87) reported similar levels of calmness compared to the two throttle condition (M = 4.83, SD = 1.30), and the mouse condition (M = 4.43, SD = 1.21), F(2,56) = 2.73, p = .07. Participants that reported lower calmness before the task, also reported lower calmness after the tasks, F(1,56) = 23.40, p < .001.

For arousal, participants in the one throttle condition (M = 4.78, SD = 1.07) reported similar arousal compared to, the two throttle condition (M = 4.53, SD = 1.24), and the mouse condition (M = 4.15, SD = 1.24), F(2,56) = 0.68, p = .38. Participants that reported lower arousal before the task, also reported lower arousal after the tasks, F(1,56) = 60.97, p <.001.

For valence, significant differences were observed between participants in the one throttle condition (M = 5.23, SD = 0.88), two throttle condition (M = 5.05, SD = 1.10), and the mouse condition (M = 4.55, SD = 1.30), F(2,56) = 3.63, p = .03. Participants that reported more positive valence before the task also reported more positive valence post the tasks, F(1,56) = 20.08, p <.001.

**Mood effects Task 3**

Differences in mood (calmness, energetic arousal and valence) were evaluated using between-subject ANOVAs. For all mood scales, the condition variable was the fixed factor, and the baseline mood ratings (mood following previous task) were used as a covariate.

For calmness, participants in the one throttle condition (M = 3.28, SD = 1.37) reported similar levels of calmness compared to the two throttle condition (M = 3.47, SD = 0.95), F(1,57) = 0.23, p = .63. Participants that reported lower calmness before the task also reported lower calmness post the tasks, F(1,57) = 11.66, p < .001.

For arousal, participants in the one throttle condition (M = 4.45, SD = 1.27) reported similar arousal compared to the two throttle condition (M = 4.48, SD = 1.29), F(1,57) = 0.23, p = .64. Participants that reported lower arousal before the task also reported lower arousal post the tasks, F(1,56) = 37.79, p <.001.

For valence, participants in the one throttle condition (M = 3.85, SD = 1.32) reported similar valence compared to the two throttle condition (M = 4.05, SD = 1.28), F(1,57) = 0.11, p = .74. Participants that reported more positive valence before the task also reported more positive valence post the tasks, F(1,56) = 9.66, p = .003.

**Supplementary materials 2 – Normality tests and plots for all tasks**

Task 1

| Condition | Shapiro-Wilk p-value |
| --- | --- |
| Mouse first | .11 |
| Mouse second | .03 |
| Throttle first | .002 |
| Throttle second | .30 |


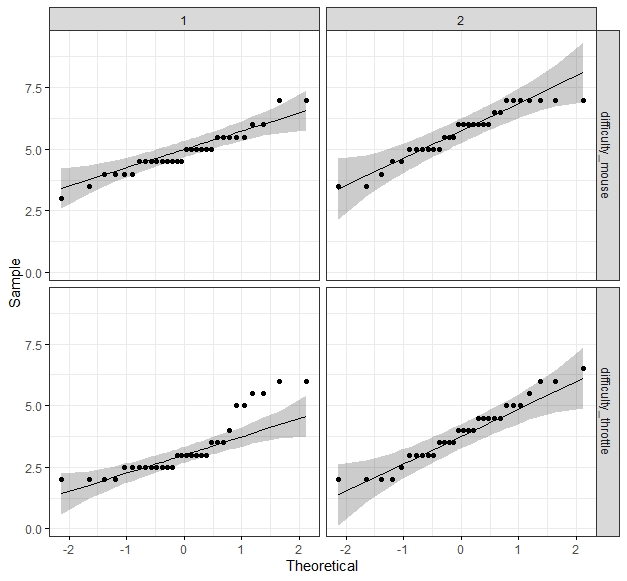


Task 1 – independence between dimensions SDs

| Condition | Shapiro-Wilk p-value |
| --- | --- |
| Mouse | 3.92E-07 |
| 2 throttles | 3.23E-15 |


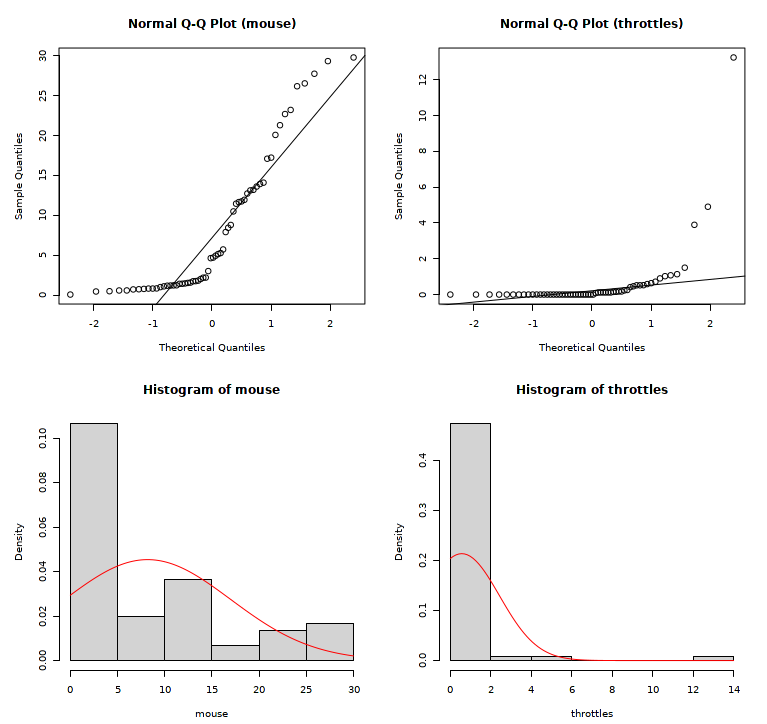


**Task 2**

| Condition | Shapiro-Wilk p-value |
| --- | --- |
| Mouse | .44 |
| 1 throttle | .02 |
| 2 throttles | .41 |


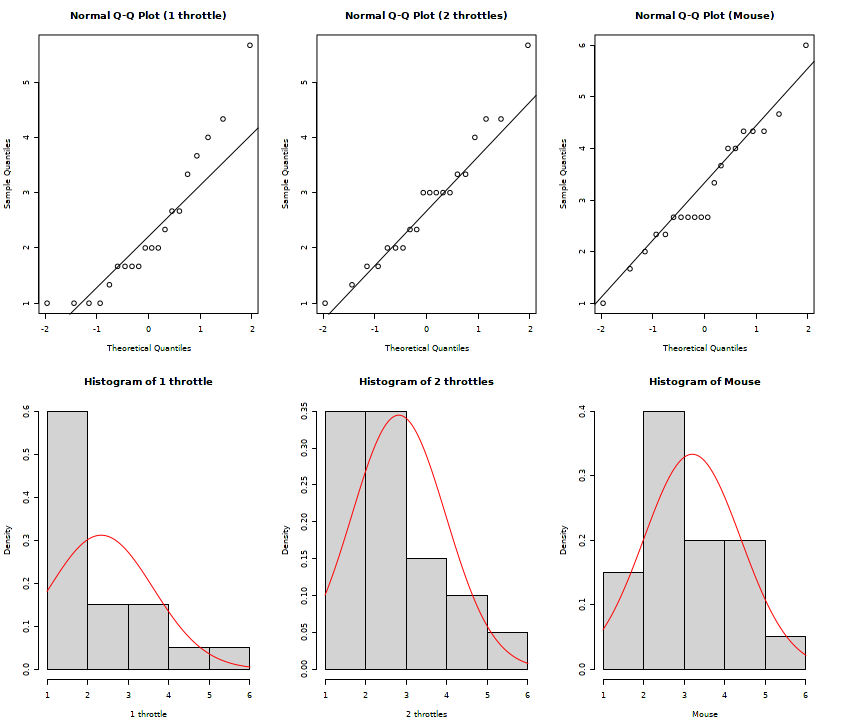


Task 3

Difficulty

| Condition | Shapiro-Wilk p-value |
| --- | --- |
| 1 throttle | .007 |
| 2 throttles | .04 |


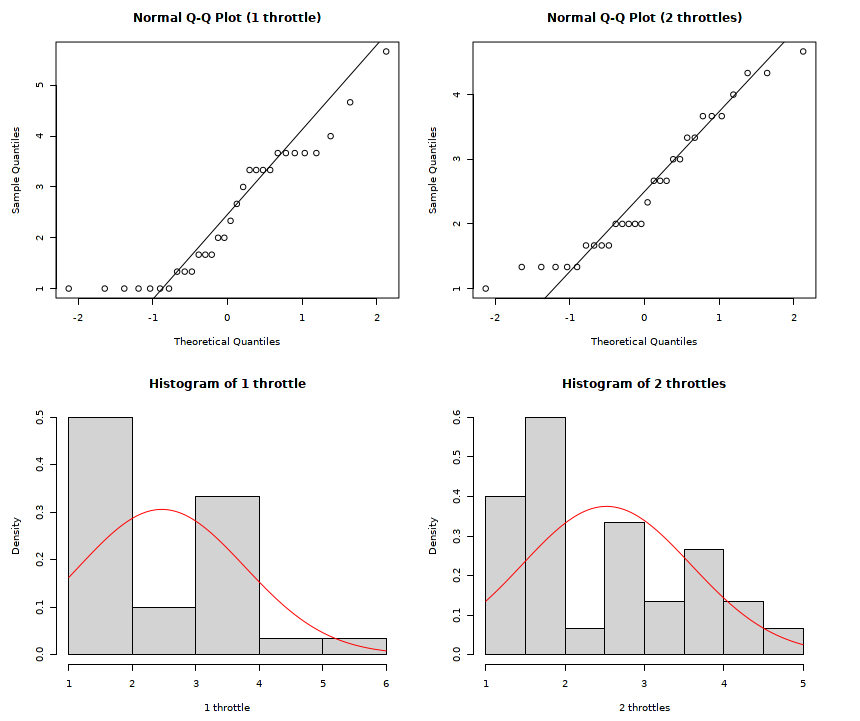


Tiredness

| Condition | Shapiro-Wilk p-value |
| --- | --- |
| 1 throttle | .002 |
| 2 throttles | .002 |


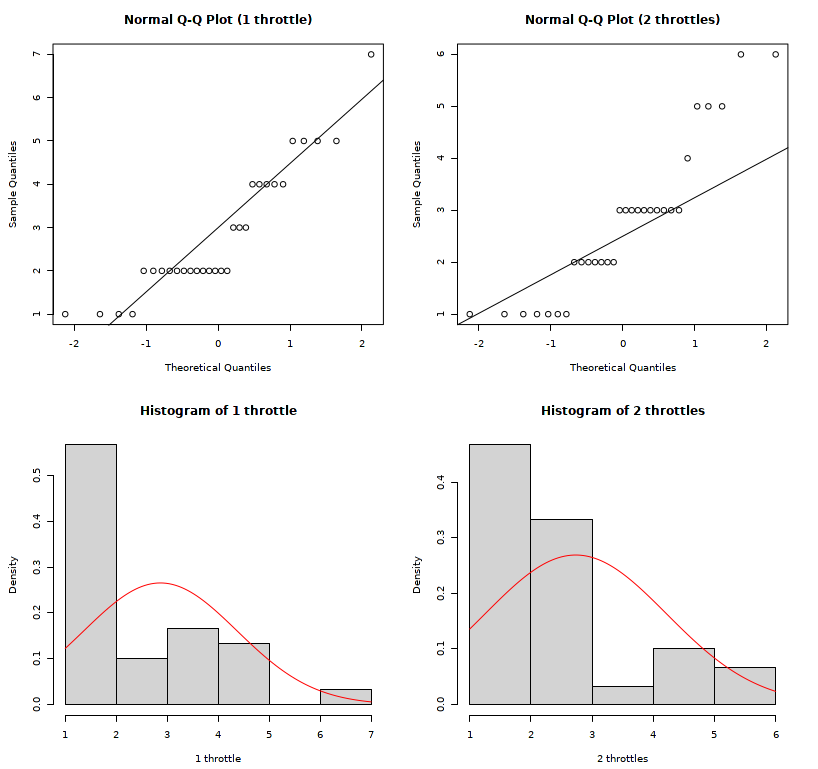


**Supplementary materials 3 - Task 3 robust percentage bend correlations**

| Table S1.  Robust percentage bend correlations between post, mean, peak and end confusion. | | | | | | | |  |
| --- | --- | --- | --- | --- | --- | --- | --- | --- |
|  | Post Confusion | | Mean Confusion | | Peak Confusion | | End Confusion |  |
| Post Confusion |  | | .72*** | | .73*** | | .69*** |  |
| Mean Confusion | .64** | |  | | .69*** | | .84*** |  |
| Peak Confusion | .70*** | | .83*** | |  | | .69*** |  |
| End Confusion | .86*** | | .77*** | | .74*** | |  |  |
| Note: * *p* <.05; ** *p* <.01; *** *p*<.001; One throttle condition correlations are displayed above the diagonal; Two throttle condition correlations are displayed below the diagonal; Correlations between all variables and post-hoc confusion ratings are based on 19 participants above the diagonal and 20 participants below the diagonal due to missing data. All other correlations are based on 30 participants. | | | | | | | |  |
| Table S2.  Robust percentage bend correlations between post, mean, peak and end interest. | | | | | | | | |
| Variable | | Mean interest | | Peak interest | | End interest | | |
| Post interest | | .40 | | .37 | | .49* | | |
| Mean interest | |  | | .70*** | | .72*** | | |
| Peak interest | |  | |  | | .48** | | |
| Note: * *p* <.05; ** *p* <.01; *** *p*<.001; Correlations between post-hoc interest ratings and all other variables are based on n = 20 due to missing data. All other correlations are based on n = 30. | | | | | | | | |

| Table S3.  Robust percentage bend correlations between post joy, sadness and being moved and sadness and joy. | | | | | | | |
| --- | --- | --- | --- | --- | --- | --- | --- |
| Variables | Sadness | | |  | Joy | | |
|  | Mean | Peak | End |  | Mean | Peak | End |
| Post sadness | .53*** | .47** | .16 |  | .21 | .37* | .41** |
| Post joy | .27 | .18 | .18 |  | .42** | .47** | .57*** |
| Post moved | .34** | .33** | .10 |  | .22 | .43*** | .42*** |
| Note: * *p* <.05; ** *p* <.01; *** *p*<.001; Correlations between post sadness and joy are based on 39 people. All other correlations are based on 60 people. | | | | | | | |
